# The Role of Psychological Distance in Influencing Pro-environmental Behavior Spread: Perceived Justice Enforceability as a Moderator

**DOI:** 10.3389/fpsyg.2020.567093

**Published:** 2020-10-28

**Authors:** Zhengquan Xu, Qinren Cao, Shuang Li

**Affiliations:** School of Management, China University of Mining & Technology, Xuzhou, China

**Keywords:** loose social system, perceived unfairness, psychological distance, pro-environmental behavior (PEB), justice enforceability

## Abstract

The social system can spread tightly coupled complex practices under the context that members of the social system do not have the shared experience that enables them to coordinate within longstanding tight formal organizations. To promote the understanding of such a process, and given the possibility for other members in the social system to cheat and adopt pro-environment behavior, we draw on the organizational justice literature and the perspective of justice enforceability, and construal level theory, to develop a conceptual model in which the impact of social members’ perceived psychological distance on their response to other social members adoption of pro-environmental behaviors (PEBs) is contingent on their perception of justice enforceability and cognitive appraisals (positive, not significant) towards other social members’ adoption of PEBs. We find that when social members perceive that the adoption of pro-environment behaviors is justice-enforceable, their cognitive appraisals of other social members’ adoption of PEBs is high, and then the more proximal the psychological distance they perceive, the stronger they will react to other social members’ adoption of PEBs. Further, they will adopt and enact such behaviors, otherwise, they would be unwilling to adopt and enact such behaviors. So, uneven perceived psychological distance of social members can harm their adoption and the spread of pro-environment behavior. We tested our model in a survey study. Results show that the proposed model is supported, and our understanding is enhanced about how social members’ willingness to adopt and spread pro-environment behavior is contingent on their perceptions of justice enforceability. This paper is comprised of five parts, of which include an introduction, a part on the theory and hypothesis, data and methods, results and discussion, and conclusion.

## Introduction

Environmental problems, especially those in developing countries, are still deteriorating, and the negative externalities generated by this deterioration have not only made developed countries suffer due to climate warming, but has also led to many other serious environmental problems, such as floods and drought (Swim et al., 2011). Research by Doran and Zimmerman (2009) has demonstrated that 97% of climatologists agree that human activities have affected climate change, which has caused serious environmental problems. Today, the governments of most countries in the world have begun to stress the importance of environmental protection, and policymakers in some countries have begun to implement policies dedicated to reducing greenhouse gas emissions (Mohai et al., 2010). Scholars have also devoted a lot of attention to addressing environmental issues and exploring measures on how to make economic and social development more environmentally friendly (De Groot and Steg, 2010). They believe that if humans can engage in pro-environmental behaviors (PEBs) in life and at work, it will be helpful in reducing the detrimental effect of human activity on the environment and improve the well-being of mankind (Wibeck, 2014).

A large number of studies on the perceived psychological distance of environmental problems have shown that people around the world are very concerned about environmental problems, but most of them do not believe that environmental problems are the most urgent problems that need to be resolved first among various problems faced by human beings (Kim and Wolinskynahmias, 2014). From the perspective of optimism (Cheng et al., 2019), people think that the risk caused by environmental changes will not bring immediate damages to them. Using their own awareness of the severity of environmental problems, they feel that such damages may soon occur to people in other regions or other countries (Kollmuss and Agyeman, 2002); From the perception of distance, they may feel that the risks brought about by environmental changes are global rather than local, and may not be able to adversely affect the area in which they live. In addition, social members may think that good environment is public goods, and protecting the environment is the responsibility of the government or other public sectors or other people, and they tend to rely on such public goods (Lorenzoni and Pidgeon, 2006), so that they may think that although environmental issues are important in their minds, they are still unwilling to adopt and spread PEBs in practice (Espeland and Kettenring, 2018). To put it differently, although the social members’ perception of the psychological distance of environmental issues affects their attitudes and behaviors toward environmental issues, the magnitude of the impact is uncertain and contingent on other factors (Gärling et al., 2003).

In this study, we address the issues of social members’ perceived psychological distance of environmental issues which affect the adoption and spread of PEBs in social systems. To put it specifically, we employ a cognitive and organizational justice perspective to explain why the adoption and spread of PEBs in social systems depend on the perceived justice enforceability of adopting PEBs and the level of cognitive appraisal (Valentine, 2017). From a cognitive perspective, there is always a gap between individuals’ cognition and their actions (Gärling et al., 2003), which can exert a very critical impact on individual behavior. When confronting environmental problems, most individuals agree that the continuing environmental degradation will do harm to all human beings, but they do not agree that it will do very serious harm to themselves (Chen, 2019). Chen’s (2015) research on self-efficacy and collective efficacy shows that people in a collectivist cultural atmosphere are more willing to adopt PEBs than the individualist cultural atmosphere in a social system because the former can foster much more of a sense of fairness than the latter can do. In addition, people in a collectivist atmosphere are more closely connected to each other (Williams and O’Reilly, 1998), this can enhance the social contacts between social members. PEBs are a new behavior for those who have not previously adopted PEBs, so their adoption of PEBs means that they will incur more of a personal cost. If they adopt PEBs but others do not, then they will feel that it is unfair (Greenberg, 1993). Thus, from the perspective of fairness, when a person is ready to adopt a new behavior, they will make a social comparison and reaffirmation to decide whether or not to adopt such a behavior (Greenberg, 1993). In a collective environment, if the psychological distance perceived by social members is proximal, then most of them voluntarily adopt PEBs, this will create a fair atmosphere which exerts positive influence on those who have not adopted PEBs (Hackman and Katz, 2010). However, in a social system, the connections between people are relatively weak, and social members tend to be more individualistic in their action orientation. The psychological distance between people is larger and more uneven, the interaction between different social members’ activities is also weaker. It is impossible to restrict social members’ behaviors by mandatory administrative means beyond legal constraints, so it is more difficult to form a fair atmosphere (Matta et al., 2017). So, we argue that there exist different mechanisms between the spread of PEBs in social systems and tightly coupled social systems. In a tightly coupled social system, due to the closer relationship between members, what members are forced to comply and are subjected to formal system constraints on what they should and should not do (Rodell and Colquitt, 2009). However, PEBs in a social system are mostly not enforced by law (Thibaut et al., 1973). Therefore, we posit that in a social system, whether social members adopt PEBs is contingent on the level of their cognitive appraisal and their fairness perception of engaging in PEBs.

We chose the Chinese society as the background to test the hypothesis in this article. In contrast to formal organizations, taking enterprise for example, Chinese society is loosely coupled, the structure of which is much more uneven. At present, China, as the largest developing country, is committed to addressing the issues of the ecological environment. The government of China not only promulgates the benefits of environmental protection but also advocates that its residents actively protect the environment. With the continuous development of China’s market economy, the government’s administrative intervention on individual behaviors of social members is becoming less evident. Whether social members adopt PEBs is largely voluntary. Therefore, it is appropriate to test the hypothesis of this study within Chinese society.

Theoretical and empirical studies of this paper contribute to the literature on psychological distance, the spread of behaviors, and organizational justice. First, our research deepens the understanding that the mechanism of impact of social members’ psychological distance on the adoption and spread of their PEBs in social systems is different from that of tightly coupled organizations.

Second, we extended the study of psychological distance to the field of spread of PEBs and found that the uneven distribution of psychological distance affects the spread of PEBs.

Finally, we introduced two key constructs of perceived fairness and cognitive appraisal into the theoretical model developed in this study, and posit that the effect of psychological distance on the positive attitude of spreading PEBs in a social system depends on the social members’ perception of fairness and cognitive appraisal of the spread of PEBs. Besides, our research can be used by the government to promote the implementation of pro-environment policies and initiatives, and therefore has important practical implications.

## Theory and Hypotheses

### Perceived Psychological Distance of Environmental Changes

The concept of psychological distance was first proposed by Beckerman (1956). He found that trade between countries is not only affected by geographic distance, but also by importers’ perceived psychological distance of potential suppliers. Later, some scholars borrowed the concept of psychological distance to study environmental problems, which proved that the public’s awareness of climate change is positively related to the severity of climate change that they perceive (Sun and Han, 2018), and Wibeck (2014) found that social members’ adoption of PEBs plays a key role in protecting the environment and countering the deterioration of the environment. Scholars in organizational behavior have demonstrated that behavior is a function of perception, and perception guides human behavior (Vanderstukken et al., 2019). The change in perception occurs before the change in behavior, and this relationship will cause a delay in the actions taken by humans to deal with the environment change (Tang et al., 2019). As environmental changes occur at a relatively slow pace and do not form a very obvious accumulative effect, after a long period of time, environmental problems will have become much more serious before humans are alarmed and make critical responses. To put it a different way, the subjectivity of psychological distance may delay human’s positive reaction to environmental changes (Milfont, 2010).

Scholars have developed many theories with psychological distance as the core concept. Among them, the construal level theory (CLT) is a relatively famous one. The CLT mainly illustrates the relationship between the psychological distance and the degree of abstract or specific human thoughts (Trope and Liberman, 2010), In other words, the subjective psychological distance perceived by the individual will have a substantial impact on its behavior in reality (Trope et al., 2007). The study of Liberman and Trope (2008) showed that psychological distance has roughly four dimensions, namely temporal distance, spatial or geographical distance, distance between the perceiver and another individuals or groups, and other dimensions that cannot be determined. In a social system, although environmental changes have had a negative effect on human society, the subjective perception of social members’ psychological distance to environmental changes lead them to believe that the problems incurred by environmental changes (such as warming and disease spread) will affect those who are far away from them, and will not have a bad influence on themselves in a short time (Spence et al., 2012). That is to say, when social members perceive that they have a large psychological distance from environmental changes, they will think that bad effects incurred by environmental changes will happen to other people or groups (Chen et al., 2018). However, when social members perceive a proximal psychological distance to environmental changes, they will be prompted to respond more positively to environmental changes (Li K. et al., 2019). To put it differently, when social members recognize or feel that the impact of environmental disasters on themselves is more proximal and visible, then their response to environmental issues will be more positive (Chu and Yang, 2018).

Although psychological distance has been extensively studied as an important academic field in the past few decades (Chen and Li, 2018), there are still many controversies on the empirical research of psychological distance among scholars (Vaccarini et al., 2017). At the micro-level, scholars have found that psychological distance affects people’s cooperation tendencies and conflicts of opinion (Zheng et al., 2019), but they do not hold consistent views on how psychological distance affects individual behavior, promotes the spread of a behavior, and makes collective behaviors emerge. In a social system, due to the bias of social members’ perception of the psychological distance of environmental changes, the government cannot achieve the goal of environmental protection by forcing all social members to adopt PEBs through compulsory means (Schultz, 2000). Previous studies rarely treated PEB as a discretionary behavior, which is determined by the social members’ willingness and depends on their fairness perception when they engage in the PEBs. Before the emergence of environmental problems, there was no so-called PEB in the classification of human social behavior, so we can even call PEB an extra-role social behavior. Research by Smith and Joffe (2013) showed that in a region with serious ecological and environmental problems, whether residents adopt or refuse to adopt PEBs is not controlled by the government, even if the government strongly advocates residents to adopt PEBs to protect the environment.

At present, residents in some countries and regions are advocated to engage in trash sorting, but few of them do this in their daily lives. This will create an unfair perception of the social members who have adopted or are about to adopt PEBs (Olivola and Shafir, 2013). There is a paucity of existing research on the above problems. In addition, the existing research has not built a consensus on how the spread of pro-environment behaviors is dependent on the perception and cognition of social members. In the face of serious environmental problems, most residents still deem that the responsibility they should bear for the consequences of environmental degradation is very insignificant and their daily lifestyle of not adopting PEBs is not enough to cause serious harm to the environment. This cognitive bias will ultimately affect the enthusiasm of them to cooperate with the government over environmental protection (Stern, 2000). Therefore, in this study, we introduce the two important concepts of fairness perception and cognitive bias to explain the contingency of social members’ spread of pro-environment behaviors affected by the perception of psychological distance.

**FIGURE 1:**
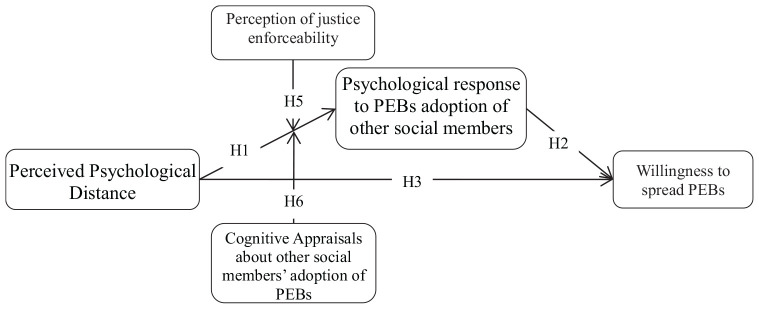
A conceptual model of social members’ psychological responses to their perceived psychological distance. Hypothesis 4 (H4) denote by the path from perceived psychological distance to willingness to spread PEBs through psychological response to PEBs adoption of other social members.

According to the definition of psychological distance, the more proximal the psychological distance to environmental problems is perceived by social members, the more likely they are to react positively to environmental changes (Dunlap et al., 1983). However, in a social system, social members are often “not concerned about issues that have no close relation to themselves,” to put it differently, social members’ perceived psychological distance to environmental problems is subjectively much more distal, promoting the spread of PEBs by shortening social members’ psychological distance to environmental changes. In this study, we draw on the CLT of psychological distance, cognitive bias, and perceived fairness to shed new light on how social members’ perception of psychological distance of environmental issues affect the spread of PEBs (Gifford, 2011). We argue that it the subjectivity of the social members’ perceived psychological distance that can be used to promote the spread of PEBs in social systems. Research on behavioral and psychological science showed that perception and cognitive biases can make the preference or bias of human behavior, and encourage humans to resolve those problems that are considered to be urgent and important (Liberman and Trope, 1998). Thus, the social members’ prejudice in the perceived psychological distance can be leveraged to influence their decision, to promote the engagement of actions that help enhance the overall interests of the social system, and to prevent the adoption of actions that are not conducive to the society. Frankly speaking, most of the people in the world have interests in environmental protection. The deterioration of the environment will hurt their interests to some degree. However, in the process of economic development, almost all countries or regions have experienced a period of severe environmental damage in the process of them evolving into developed economies. Most of developing countries consciously or unconsciously prioritize economic development over environmental protection before they become a developed country. In the same vein, social members’ perceived psychological distance toward the environmental changes is also biased. In addition, in a social system, the social members’ perception of the psychological distance of environmental changes will affect their psychological response to other social members’ adoption of PEBs, and will further have a critical impact on whether they themselves adopt PEBs or not. Further research shows that the magnitude of the impact of the psychological distance on psychological response is contingent on the level of social members’ cognitive appraisal and their perception of justice enforceability (Lea, 1994). In a region or country with a relatively high level of education, the social members’ perception of the psychological distance of environmental problems usually invokes a much stronger psychological response to PEBs and further enhances their willingness to adopt PEBs (Lorenzoni and Pidgeon, 2006). Similarly, social members will also be more willing to adopt PEBs if the governmental environmental policies and initiatives are perceived to be fair and fairly enforceable. Because, if the environmental protection initiatives are perceived by social members to be fair and fairly enforceable in reality, they will not feel deceived when adopting PEB themselves. Besides, social members engaging in PEB need to pay the extra cost and consume extra resources, so when they are adopt PEB, but others do not adopt it, then they will feel that their extra effort has been for nothing, and will have a sense of injustice (Colquitt, 2004). In this unfair atmosphere, the influence of social members’ perception of psychological distance on social members’ psychological responses to other people who do not adopt PEBs will be weakened, thereby further reducing their willingness to adopt PEBs (Colquitt et al., 2001). Following Klöckner and Blöbaum (2010), and based on the above discussion, we can develop the conceptual model of this study (see Figure 1).

### Relationship Between PEBs, Social Members’ Perceived Psychological Distance of Environmental Changes, and Their Psychological Responses to Other Social Members’ PEBs

Drawing on signaling theory, Harmon argued that social members in a social system can receive a lot of information about PEBs from different sources, some of which are issued by the government, some by the news media, and some by their own friends (Harmon, 2019). When social members are faced with so much information, they will rank the importance of this information according to their perceived cognitive distance and time distance, so naturally they will not treat the information fairly. Information that supports their own beliefs, or is beneficial to them, is usually given priority. In so doing, the information received will be filtered, they will consciously or unconsciously avoid messages that are in conflict with their predispositions (Spence, 1973). Therefore, in the social system, psychological distance is considered to be a key factor affecting behavior (Puchalska-Wasyl, 2018). For an American, when they face two problems, the plague in Africa and their friend’s toothache, if they are required to rank the importance of the above two things, they are likely to put much more weight on the latter over the former. According to the CLT theory, Americans perceived the psychological distance of their friends’ toothache to be much more proximal than that of the African plague. If they are asked to take immediate action, they will go to send their friend to the dentist as soon as possible, instead of running to the Red Cross to donate money to help Africans fight the plague. In a social system, not only is the physical distance between people uneven, but the psychological distance between people is also uneven, so social members’ behavior will have a greater impact on their friends than strangers (Kivetz and Tyler, 2007).

In a similar vein, social members’ prejudice against the perceived psychological distance of environmental changes will form a filtering effect of cognition and perception. For policymakers in China, they will tend to allocate more resources to promote economic development than to protect the environment. For the individual social member, the deterioration of the environment that can do damage to individual interests (wellbeing) and can also form a bad public effect. An old Chinese proverb says, “the tall person will hold up the sky when it collapses,” and the individual will also free-ride on environmental protection, because he deems that environmental degradation is hurting other people more than himself, or the damage he has suffered is minimal, so he will feel that it is reasonable that the responsibility for protecting the environment should be borne by others or the government (Ambos et al., 2019). However, if social members often have contact with friends or neighbors who are negatively affected by environmental problems in their daily lives, then they will make a more positive evaluation when other social members adopt pro-environment behaviors (Safari and Chetty, 2019). Using the same logic, when a social member who does not adopt PEB faces increasingly serious environmental problems, if his friends, not strangers or distant foreigners, have adopted pro-environment behaviors, they will react more positively to their friends’ adoption than to strangers’.

This leads us to predict:

Hypothesis 1. The more proximal social members’ perceived psychological distance of environmental changes is, the more positive their response is to other social members’ adoption of pro-environment behaviors.

(*Note: below, we use the reverse indicator to measure the psychological distance, so this effect in the later statistical analysis is positive.*)

### The Effect of Social Members’ Psychological Response to Others’ Adoption of PEBs on Them Spreading PEBs

In a social system, if social members perceive a proximal psychological distance to environmental issues, and most of the other social members have adopted PEBs, they will respond more positively by spreading positive word-of-mouth to the person who is engaging in PEBs (Swan and Oliver, 1989), and be willing to donate to environmental protection organizations (Nordman and Tolstoy, 2014). However, social members’ positive reaction to PEBs is not the ultimate goal of a social system. We expect that social members’ positive attitudes toward others’ adoption of the PEBs can be transformed into their own PEBs, then they will voluntarily spread PEBs to others who do not adopt PEBs (Bord et al., 1998). In other words, we hope that they not only think that PEB is very important, but also engage in it in reality, and are willing to spread, through social contact, PEB to other social members who have not adopted the PEB.

In reality, the government strives to resolve increasingly serious environmental problems by the implementation and propaganda of environmental protection policies which can inspire social members to form a positive attitude toward PEBs. It is not the ultimate goal of the government to change social members’ attitudes toward environmental issues, the government wishes all social members to engage in PEBs in their daily life or work. Obviously, it is not enough to only rely on the power of the government to solve environmental problems. The government also needs to mobilize all social members to participate in the work of environmental protection, and make PEBs spread among different social members through extensive social contacts. Therefore, creating a positive psychological response to environmental protection issues is different from making social members adopt and spread PEBs. In the social system, all human social behavior can spread through social contact. PEB, as a kind of social behavior, can also spread through social contact between people (Van Kleef, 2009).

Pro-environmental behavior is a strong predictor of whether social members will take action to protect the environment. Through social contact, we expect that social members’ positive psychological response to others’ adoption of PEBs will eventually prompt them to actively adopt PEBs. When more and more social members adopt PEBs, the cost of implementing environmental protection policies will not only be reduced, but also the spread of PEBs through social contact will be promoted. The intention to adopt or spread PEBs is a behavioral construct that is influenced by the attitudes and emotions of social members toward PEBs (Avloniti and Filippaios, 2014). The positive attitudes of social members to adopt and spread PEBs are mainly displayed through the social members’ lifestyle changes in reality, advocacy and participation in various environmental protection actions (Piazza and Jourdan, 2018). For those social members who are willing to adopt and spread PEBs, they will make positive statements on the adoption of PEBs to those they contact. Willingness is a key predictor of behavior (Vlachos et al., 2017). The same logic holds true that if social members’ willingness to adopt and spread PEBs increases, the possibility of their adopting and spreading PEBs will increase too. Drawing on the theory of spread and social comparison theory, we posit that social members’ positive psychological response to others’ adoption or spread of PEB will enhance the possibility of themselves adopting PEB in reality in several ways.

First, from the perspective of the spread of behavior, both social influence and social contact can have an impact on the spread of behavior (Theeke et al., 2018). In other words, social contact and social influence can promote the spread of behavior. So, social members can spread PEBs through social contacts. For example, an individual may adopt PEBs because relatives and friends they often contact have adopted PEBs. On the other hand, social members’ adoption of PEB may also be due to social influence. Although there is no so-called social contact between most of people in a social system, when more and more social members adopt PEBs, the social pressure generated by the spread of PEBs grows on the individuals who have not adopted PEBs. Such pressure will gradually force them to adopt PEBs. Dunlap et al. (1983) showed that some tourists from a country with serious environmental problems will quickly change their existing behavior patterns and adopt PEB when they come to tourist destinations where environmental protection is very good. Thus, both social influence and social contact can prompt social members to adopt and spread PEBs. Social contact can promote the spread of PEB through individuals’ point-to-point interaction, while social influence can promote the spread of PEB through group-to-individual interaction.

Second, social members’ psychological responses to others’ adoption of PEBs can be incurred by social comparisons. The social system has the function of filtering and assimilating its members’ behaviors. Social members will make a dynamic comparison of behaviors they want to engage in. When they feel that their behaviors are different from the behaviors of most people engaged, then they return their deviated behavior to the existing social behavior trajectory through social comparisons (Selenko et al., 2017). Said differently, the behavior of social members in a social system must always conform to social norms, and evolve with the evolution of social norms. When most people have adopted PEBs, those who have not adopted PEBs will find their behavior deviates from the norm through social comparison and correct their behaviors according to mainstream social norms. So, we hypothesize:

Hypothesis 2. Social members’ positive psychological reaction to others’ adoption of PEBs has a direct positive effect on their willingness to spread PEBs.

When the social members respond positively to others’ adoption of PEBs, they tend to invest more effort into spreading PEBs through social contact. Research on psychological distance showed that the more proximal the social members’ perceived psychological distance of environmental problems is, the more positive their psychological response is to others adopting PEB. To put it another way, when social members feel that the environmental degradation is very serious, and their close relatives and friends all begin to adopt PEBs, their strong positive psychological reactions to PEBs will be stirred, then their willingness to adopt PEBs will increase too. So, on the basis of hypothesis 1 and 2, we can make the following hypothesis:

Hypothesis 3. The perceived social members’ psychological distance of environment changes also has a direct positive influence on their willingness to spread pro-environmental behavior.

### Indirect Effect of Psychological Distance on the Willingness of Social Members to Spread PEBs

Furthermore, social members’ positive response to others’ adoption of PEBs is the leading indicator of their willingness to spread PEBs, and social members’ perceived psychological distance of environmental issues is the leading predictor that can be used to judge if they react positively when other social members adopt PEBs (Weisner and Sutton, 2015). Therefore, we can speculate that the changes in the social members’ perceived psychological distance of environmental issues can first exert an impact on their positive psychological reactions to others’ adoption, then on their own willingness to spread PEB. Said differently, social members’ positive psychological reactions may mediate the impact of psychological distances on willingness to spread PEB.

In a social system, when the environmental problems perceived by the social members become more serious, more social members who actively address the environmental problems will form social pressures on those who have not acted yet, and such social pressures can stir their willingness to engage in environmental protection (Trautmann, 2019).

This leads us to propose the following hypothesis:

*Hypothesis 4. Social members’ psychological reaction to others’ adoption of PEBs mediates the effect of their perceived psychological distance on their willingness to spread PEBs*.

### The Moderating Effect of Cognitive Appraisals and Justice Enforceability

As such, we have discussed the reasons why social members’ perceived psychological distance affects their psychological responses to others’ adoption of PEBs, and how their psychological responses to others’ adoption of PEBs affect their willingness to spread PEB. Below, we will focus on the interaction between social members’ psychological distance, perception of justice enforceability, and cognitive appraisals. Thus far, we have argued that the perception of psychological distance affects social members’ positive psychological response to others’ adoption of PEBs, the latter in turn affects the willingness of them to spread PEBs. Because social members’ perception of psychological distance toward environmental changes can enhance their psychological response to others’ adoption of PEBs, so we posit that the impact yielded in the process from social members’ psychological distance to their willingness to spread PEB relies on social members’ perception of justice enforceability and cognitive appraisals. It will be of significance in theory and management practice to determine under what conditions impact produced in the above process will be strengthened or weakened. The studies of Kruglanski (1989) and Van Kleef et al. (2004) and others showed that the impact yielded in the above process depends largely on the social members’ cognitive appraisals and their perception of justice enforceability toward PEBs. That is to say, when social members have a higher level of cognitive appraisal toward environmental issues, and feel that government environmental protection policies or initiatives can be implemented fairly, the effect of social members’ perceived psychological distance on their positive psychological reactions to others’ adoption of PEBs will be magnified.

Following Valentine (2017), we introduce the idea of justice enforceability, “defined as the perception that authorities can act fairly, given the potential for other people to cheat” (Valentine, 2017). In a social system, the social members’ perceptions of justice enforceability of PEBs were focused on whether the adoption of PEBs could be cheated. Because some social members may verbally promise that they will adopt PEBs, but do not put PEBs into action. When this occurs, the impact of the social members’ perception of psychological distance on their positive psychological response to others’ adoption of PEBs will change too. Thus, we posit that the effect of social members’ perceived psychological distance on their positive psychological reactions is expected to become stronger as social members’ perceived justice enforceability toward the execution of environmental protection initiatives increases. To put it differently, if social members deem that the government’s environmental protection initiatives or environmental protection policies can be fairly enforced in reality, their positive psychological response to others’ adoption of PEBs will be amplified, and their willingness to spread PEB is also enhanced.

Cognitive appraisals are inferences that a social member draws about the other social members’ true feelings and intentions of adopting PEBs. Cognitive appraisals require social members to make inferences about the intention of PEBs’ adoption by other social members, which guide the former’s behaviors by providing contextually relevant information about the latter. Thus, drawing on the study of Valentine (2017), we further argue that when social members find that they need to pay extra costs or consume extra resources for adopting altruistic extra-role behaviors, they will become more cautious and reaffirm whether it is fair for them to expense the extra cost or resources. As environmental problems begin to deteriorate, those social members with low cognitive appraisals will deem that the deteriorating environmental problems can do little harm to themselves, they will be indifferent to other people who are engaging in PEB (Chen, 2019). On the contrary, for people with high cognitive appraisals, they will deem that environmental deterioration will eventually make everyone a victim, and they will have a more positive psychological response to others who are engaging in or have adopted PEBs. In this case, whether social members have a positive psychological response to others who are engaging in PEB will largely depend on their own cognitive appraisals. So, we predict that cognitive appraisal and perceived justice enforceability moderate the relationship between social members’ perceived psychological distance and their psychological responses to others’ adoption of pro-environment behaviors.

Therefore, we can hypothesize:

***Hypothesis 5. Social members*** *perceived justice enforceability moderates* ***the impact of*** *their perceived psychological distance on their psychological responses to others’ adoption of pro-environment behaviors.* ***As the social members perceive that environmental protection policies can be more fairly enforceable, the impact of their perceived psychological distance to environmental issues on*** *their psychological responses to others’ adoption of pro-environment behaviors* ***will be weakened.***

***Hypothesis 6. Social members*** *cognitive appraisals moderate* ***the impact of*** *their perceived psychological distance on their psychological responses to others’ adoption of pro-environment behaviors.* ***As the social members’*** *cognitive appraisals increase*, ***the impact of their perceived psychological distance to environmental issues on*** *their psychological responses to others’ adoption of pro-environment behaviors* ***will be weakened.***

(*Note: we use the reverse indicator to measure the psychological distance, so the above moderating effect in the later statistical analysis is positive*).

## Data and Methods

### Sample

Urban residents showing the initiative to classify domestic waste are considered to be engaging in PEBs. At present, most urban residents in China are faced with the problem that their city is besieged by garbage, which has seriously damaged the ecological environment they are living in. In addition, most cities in China use landfills to dispose of garbage, which not only wastes a lot of land resources, but may even cause very long-lasting pollution problems of groundwater resources. What is more, some cities have already faced a situation where there is no land to bury garbage. Some cities besieged by garbage also face the issue of how to sustain economic development and reduce negative impacts on the daily lives of residents. So, when urban residents see the bad ecological environment in their urban-rural linking area, will they perceive a more proximal psychological distance to environmental degradation and respond positively to environmental protection? In addition, the Chinese government is now encouraging urban residents to classify garbage, and garbage classification is regarded as an important measure for handling the deterioration of environmental problems by the Chinese government. At present, a small number of cities in China have begun to carry out garbage classification, but the effect is current unsatisfactory. Externalities shaped by garbage classification are not significant. In particular, cities that have carried out garbage classification have not formed a good spillover effect on those cities that have not implemented garbage classification. Some cities have already built up infrastructure for garbage classification, why are their residents still unwilling to engage in garbage classification? In order to test the hypotheses proposed in this study, we mainly collected data about urban residents’ garbage classification.

This study mainly selected urban residents in three different cities in China as the respondents of the questionnaire distributed. In mid-April, Surveys were distributed to 330 respondents who live in these 3 different cities, namely Xuzhou in Jiangsu province (A), Jining in Shandong province (B), and Huaibei in Anhui province (C), 110 for each city. Respondents were a random sample of urban residents who have lived in these 3 different cities for many years. The first author and two research assistants were responsible for distributing and collecting questionnaires in Huaibei, Anhui, and then the two research assistants went to Xuzhou, Jiangsu and Jining, Shandong to distribute and collect questionnaires. The survey lasted approximately 6 weeks. We usually distributed questionnaires to local residents in shopping malls or squares. Before issuing the questionnaires, we usually asked the residents how many years they have lived in the city. If they have lived there for more than 3 years, we would continue the investigation.

Finally, 234 respondents completed the questionnaires. The number of questionnaires recalled from the three cities Xuzhou, Jining, and Huaibei were 91, 67, and 76, respectively. The overall response rate was 70.91%. An abbreviated 5-point Likert scale, which contained no more than 25 questions, was used for improving the quality of the survey and data collection. In so doing, respondents could complete the survey within 15 min.

The average age of respondents was 35.53 years (sd = 10.56), and other demographic data are presented in Table 1.

**TABLE 1:** Demographic data of the respondents (*N* = 234).

Demographic characteristics	Terms	Frequency	Percentage
Sex	Male	107	45.7
	Female	127	54.3
Age	≤35	55	23.5
	36–45	62	26.5
	46–55	63	26.9
	≥56	54	23.1
Education	Other	10	4.3
	Below bachelor’ degree	84	35.9
	Bachelor’s degree	95	40.6
	Master’s degree	45	19.2
Region	Xuzhou (A)	91	38.9
	Jining (B)	67	28.6
	Huaibei (C)	76	32.5

### Measures

#### Psychological Distance

Given the large number of items measuring psychological distance, following Williams and Oboyle (2008), we modeled the construct with four items selected from its four different dimensions. The items, adapted from Li S. et al. (2019) and Spence et al. (2012), were on a five-point Likert scale (1 = strongly disagree, 5 = strongly agree). Here, we place particular emphasis on the relationships between social members’ psychological distance and their psychological response to others’ adoption of PEBs. We use negative effects to measure the psychological distance. Doing so will turn the negative relationship into a positive one. The item measuring spatial or geographical distance is: “I feel that the place where I live has been negatively affected by environmental changes”; the item measuring social distance is: “I feel that the lives of people around me are negatively affected by environmental changes”; the item measuring temporal distance is: “I think in recent years my life has been more negatively affected by environmental changes.” The item measuring uncertainty of social members’ perception of environmental changes is: “I am more and more confident about the negative results brought about by environmental changes.” Cronbach’s α for the scales was 0.973.

#### Social Members’ Psychological Reaction to Others Adoption of Pro-environmental Behaviors

Here, we drew on and adapted the scales developed by Hennig-Thurau et al. (2006), Tsai and Huang (2002) to measure social members’ psychological reaction to others’ adoption of PEBs. The items are on a five-point Likert scale (1 = strongly disagree, 5 = strongly agree) and include: “I wish to adopt pro-environmental behaviors when more and more people around me have adopted pro-environmental behaviors”; “I feel that environmental protection is really important when I frequently exposed to the propaganda of the environmental protection”; “I really enjoyed interacting with social members who have adopted PEBs.” Cronbach’s α for the scales was 0.984.

#### Social Members’ Willingness to Spread Pro-environmental Behaviors

Following Groth et al. (2009), we created a three-item scale to measure social members’ willingness to spread PEBs. The 5-point Likert scale (1 means completely disagree, and 5 means completely agree) included: “I am willing to actively spread PEBs because the protection of the environment is very important for human beings”; “Next time if I see someone doing damages to the environment, I am willing to stop them”; “I am willing to recommend pro-environmental behaviors to others.” Cronbach’s α for the scales was 0.962.

#### Social Members’ Perception of Justice Enforceability

In this study, we measured social members’ perception of justice enforceability by assessing their perception of justice enforceability of government environmental policy. Following Rupp et al. (2013), we created a four-item scale to measure social members’ perception of justice enforceability of government environmental policy: “I feel that the existing environmental protection policies can fairly promote everyone’s willingness to protect the environment”; “I feel that the existing environmental protection policies do not just make a few people adopt pro-environmental behaviors”; “I feel that if other people have begun to engage in pro-environmental behaviors, I should also adopt pro-environmental behaviors”; “I feel that if I have adopted pro-environmental behavior, others should also adopt pro-environmental behavior” (1 means completely disagree, and 5 means completely agree). Cronbach’s α for the scales was 0.972.

#### Social Members’ Cognitive Appraisals of Others’ Engagement in Pro-environmental Behaviors

Social members’ cognitive appraisal of others’ engagement in PEBs was measured by their assessment of the positive value of others’ adoption of PEBs. Following Wang et al. (2017), we used a three-item scale to assess social members’ cognitive appraisal of others’ engagement in pro-environmental behaviors: “I’d say that social members who adopt pro-environmental behaviors really love the beautiful environment”; “I think that those social members are really voluntary to adopt pro-environmental behaviors”; “I’d say that purpose of those social members who adopt PEBs is really for protecting the environment.” Cronbach’s α for the scales was 0.970.

### Control Variables

In the literature of psychological distance and cognitive science, scholars have proved that people’s education, age, and region will affect their psychological response to others’ adoption of PEBs. We control for the academic background of the respondents, mainly because respondents with higher education will be more susceptible to the severity of environmental problems. Their psychological reaction will be more positive when they see others’ adoption of pro-environment behaviors. We code a 1 for “Other,” a 2 for “Below bachelor’s degree,” a 3 for “Bachelor’s degree,” and a 4 for “Master’s degree.” We control for the age of the respondents, because the younger generation in China will be more concerned about environmental changes. We code a 1 for age “≤35,” a 2 for “36–45,” a 3 for “46–55,” and a 4 for “≥56.” We control for the region that the respondents live in, because city managers have different propaganda on environmental protection, so that respondents living in different cities have different attitudes toward PEB. We code a 1 for “Huaibei,” a 2 for “Jining,” and a 3 for “Xuzhou.”

### Validity Analysis

We evaluated the factor structure of the measures through a confirmatory factor analysis (CFA) of the latent variables in our model: p*erceived psychological distance*, p*sychological response to PEBs adoption of other social members*, w*illingness to spread PEBs*, p*erception* of *justice enforceability*, and *cognitive appraisals about other social members’ adoption of PEBs*. Usually, the threshold of factor loading needs to exceed 0.6, and the threshold of scale reliability needs to be greater than 0.7 (Campbell and Fiske, 1959). The standardized loadings in the measurement model exceed 0.6 and load on their respective factors (see Table 2). The hypothesized five-factor model displayed good fit, when individual scale items were loaded on separate first-order latent factors (χ^2^(109) = 487.02; *p* = 0.000, RMSEA = 0.099; SRMR = 0.05; CFI = 0.954; Hu and Bentler, 1999).

**TABLE 2:** Results of the confirmatory factor analysis.

Variables	Items	Standardized loadings	SE	*p*
*Perceived psychological distance*	x11	0.955	0.019	***
	x12	0.972	0.014	***
	x13	0.976	0.013	***
	x14	0.973	0.014	***
*Psychological response*	x21	0.964	0.012	***
	x22	0.986	0.008	***
	x23	0.98	0.009	***
*Willingness to spread PEBs*	x31	0.952	0.015	***
	x32	0.948	0.013	***
	x33	0.944	0.014	***
*Perception* of *justice enforceability*	x41	0.954	0.012	***
	x42	0.957	0.011	***
	x43	0.945	0.013	***
	x44	0.933	0.015	***
*Cognitive appraisals*	x51	0.944	0.014	***
	x52	0.961	0.012	***
	x53	0.965	0.011	***

*n* = 234, For ease of discussion, with X1 designating *perceived psychological distance*, X2 designating *psychological response to PEBs adoption of other social members*, X3 designating *willingness to spread PEBs*, X4 designating *justice enforceability*, and X5 designating *cognitive appraisals*. ***p < 0.01.

In order to rule out the possibility of common method bias accounting for these results, we also tested a model with two latent factors, one of the latent factors contains the whole items measuring *perceived psychological distance*, *psychological response to PEBs adoption of other social members*, and the other latent factors contains the whole items measuring *perception* of *justice enforceability* and *cognitive appraisals*. The fit of this model is poor (χ^2^154 = 710.32; RMSEA = 0.17; SRMR = 0.16; CFI = 0.955). In the same vein, we also tested the model with three or four latent factors, the fits of these models are all worse than the five-factor model.

## Results

The means, standard deviations, correlations of the variables, and reliability estimates are shown in Table 3. *Psychological response* is positively related to *perceived psychological distance* (r = 0.648, *p* < 0.01) and *willingness to spread PEBs* (*r* = 0.646, *p* < 0.01), *perceived psychological distance* is positively related to *willingness to spread PEBs* (*r* = 0.825, *p* < 0.01).

**TABLE 3:** Descriptive statistics, correlations, and reliabilities.

	Mean	SD	1	2	3	4	5	Age	Sex	Education	Region
X1	3.33	1.42	(0.973)								
X2	2.43	1.22	0.648**	(0.984)							
X3	2.5	1.04	0.825**	0.846**	(0.962)						
X4	2.31	1.05	0.718**	0.786**	0.630**	(0.972)					
X5	2.24	1.11	0.681**	0.750**	0.616**	0.668**	(0.97)				
Age	35.53	10.56	-0.979**	-0.974**	-0.855**	-0.774**	-0.734**				
Sex	0.42	0.49	-0.014	0.009	-0.003	0.020	0.031	0.010			
Education	2.58	0.7	0.083	0.060	0.130^∗^	0.099	0.103	-0.087	-0.049		
Region	2.01	0.82	0.955**	0.940**	0.774**	0.686**	0.633**	-0.946**	-0.013	0.047	

Internal reliabilities (Cronbach’s alphas) for the constructs are provided in parentheses on the diagonal. *p < 0.05; **p < 0.0.01.

Below, we tested these hypotheses about (conditional) indirect effects by relying on Hayes’s (2013) PROCESS macro in SPSS.

After we controlled for education, age, and region, the results of the hypothesis testing using the Model 9 of the process (Hayes, 2013) are reported in Tables 4–6.

**TABLE 4:** Path coefficients of the conceptual model.

	Consequent							
	
	X2				X3			
		
Antecedent		Coeff	SE	p		Coeff	SE	p
X1	α	0.275	0.034	0.000	$c_{1}^{\prime }$.	163	0.080	0.043
X2					$c_{2}^{\prime }$.	542	0.093	0.000
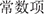	i_*X2*_	0.948	0.109	0.000	i_*X3*_	0.648	0.0966	0.000
	$R_{X\,2}^{2}$0.9574				$R_{X\,3}^{2}$0.7210			
	*F*_*X*2_(5,228)1023.859,p < 0.05				*F*_*X*3_(2,231)298.471,p < 0.05			

*α denotes the path from *perceived psychological distance* to *psychological response*; $c_{1}^{\prime }$enotes the path from *psychological response* to *willingness to spread PEBs*; $c_{2}^{\prime }$enotes the path from *perceived psychological distance* to *willingness to spread PEBs*.*

**TABLE 5:** Index of moderated mediation (X1→X2→X3).

X4	X5	Effect	BootSE	BootLLCI	BootULCI
1.000	1.000	0.272	0.057	0.154	0.378
1.000	2.000	0.303	0.073	0.161	0.450
1.000	3.667	0.357	0.113	0.157	0.595
2.000	1.000	0.362	0.073	0.211	0.494
2.000	2.000	0.394	0.078	0.228	0.532
2.000	3.667	0.447	0.106	0.237	0.653
3.500	1.000	0.497	0.109	0.278	0.708
3.500	2.000	0.529	0.104	0.309	0.718
3.500	3.667	0.583	0.114	0.336	0.777

**TABLE 6:** Results of the fit of the conditional process model.

	coeff	se	t	p	LLCI	ULCI
constant	0.948	0.109	8.686	0.000	0.733	1.163
X1	0.275	0.034	8.104	0.000	0.208	0.342
X4	–0.338	0.190	–1.779	0.077	–0.712	0.036
Int_1	0.167	0.060	2.801	0.006	0.049	0.284
X5	–0.279	0.190	–1.469	0.143	–0.653	0.095
Int_2	0.059	0.059	0.994	0.321	–0.058	0.176
age	–0.0681	0.0085	–8.0618	0.0000	–0.0848	–0.0515
edu	–0.0314	0.0196	–1.5998	0.1110	–0.0700	0.0073
region	0.3604	0.0597	6.0383	0.0000	0.2428	0.4781
*R*^2^ = 0.957, *F*(5, 228) = 1023.859, *p* = 0.000 Int_1 = X1 × X4, Int_2 = X1 × X5						

*X1 denotes *perceived psychological distance*, X2 denotes *psychological response to PEBs adoption of other social members*, X3 denotes *willingness to spread PEBs*, X4 denotes *perception* of *justice enforceability*, and X5 denotes *cognitive appraisals*.*

### Direct Effects

We used the Hayes’s (2013) PROCESS procedure (model 14, default settings) for testing hypotheses in this study. The results of the model fit are shown in Table 4.

We estimated 95% confidence intervals for the direct effects by bootstrapping 5,000 samples, results are reported in Table 4. As Table 4 indicates, there is a positive correlation between social members’ perceived psychological distance and their psychological responses to other people’s adoption of PEB (because *we use negative effects to measure the psychological distance*). The regression coefficient between the above two variables is 0.275 (β = 0.275, *p* < 0.05), and the 95% confidence interval is [0.208, 0.342] that does not straddle zero, so zero can be confidently ruled out, thus Hypothesis 1 was supported. To put it differently, the more proximal the psychological distance that the social members in a social system perceive, the stronger they will positively react to others’ adoption of PEBs. Social members’ psychological response to others’ adoption of PEBs exhibit a positively effect on their willingness to spread PEBs (β = 0.163, *p* < 0.05), supporting Hypothesis 2. We found that the direct effect of social members’ perceived psychological distance on their willingness to spread PEBs was significant and positive (β = 0.542, *p* < 0.05), providing support for Hypothesis 3.

Through the test of Hypotheses 1–3, we observed that social members’ perceived psychological distance not only exhibited a direct effect on their psychological response to others’ adoption of PEBs, but also exhibited a direct effect on their willingness to spread PEBs. Therefore, if social members’ psychological responses to other people’s adoption of PEB mediates the influence of their perceived psychological distance (X1) on their willingness to spread PEB (X3), the mediating effect is just partial. Below, we will further examine whether the indirect effect of perceived psychological distance (X1) on the willingness of social members to spread PEB is significant or not.

### Indirect Effect

We estimated 95% confidence intervals for the indirect effects by bootstrapping 5,000 samples, results are reported in Table 5.

**FIGURE 2:**
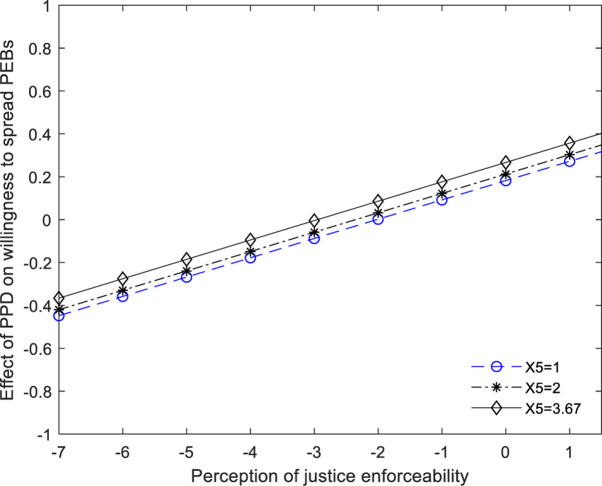
A visual representation of the conditional indirect (values of perception of justice enforceability is 1, 2, and 3.67, respectively). PPD, perceived psychological distance, X5 = cognitive appraisals.

**FIGURE 3:**
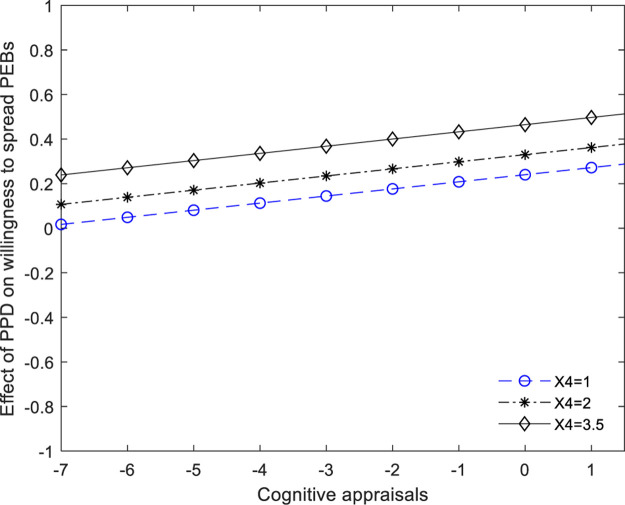
A visual representation of the conditional indirect (values of perception of *cognitive appraisals* is 1, 2, and 3.5, respectively). X4 = perception of justice enforceability.

As Table 5 and Figure 2 indicate, at three different values for *cognitive appraisals* corresponding to the 1.000, 2.000, and 3.667, all confidence intervals [BootLLCI, BootULCI] do not included zero. So, we could claim that social members’ *psychological response to PEBs adoption of other social members* just partially mediated the effect of their *perceived psychological distance on their willingness to spread PEBs.* Hypothesis 4 was supported. In addition, we can see from Figures 2, 3 that in contrast to the condition of justice enforceability, when the social members’ cognitive appraisals gradually increased, the effect of changes of their perceived psychological distance on their willingness to spread PEB was much weaker.

### Test of the Moderating Effects

We estimated 95% confidence intervals for the moderating effects by bootstrapping 5,000 samples, results are reported in Table 6.

The regression coefficient of “perceived psychological distance × *perception* of *justice enforceability*” is positive and significant (β = 0.167, *p* < 0.05), supporting Hypothesis 5. Therefore, we could claim that the *perception* of *justice enforceability* positively moderated the impact of social members’ perceived psychological distance (X1) on their psychological responses to others’ adoption of PEBs.

Besides, the regression coefficient of “perceived psychological distance × *cognitive appraisals*” is positive but not significant (β = 0.059, *p* = 0.321), the confidence interval [-0.058, 0.176] includes zero. Therefore, we could not definitely claim that the *cognitive appraisals* positively moderated the impact of social members’ perceived psychological distance (X1) on their psychological responses to others’ adoption of PEBs. So, Hypothesis 6 was not supported.

For the covariates “age, edu, and region,” we can see from results of the fit of the conditional process model that the effect of social members’ education on their psychological responses to other people’s adoption of pro-environment behaviors is not significant (β = -0.0314, *p* = 0.1110). Age of social members have a negative effect on their psychological responses to other people’s adoption of pro-environment behaviors (β = -0.0681, *p* < 0.05). That is, the older the social members are, the weaker the positive psychological reactions they would make to other social members’ adoption of pro-environment behaviors. Social members in economically developed areas will have a more positive psychological response when facing other social members’ adoption of pro-environment behaviors (β = 0.3604, *p* < 0.05).

**FIGURE 4:**
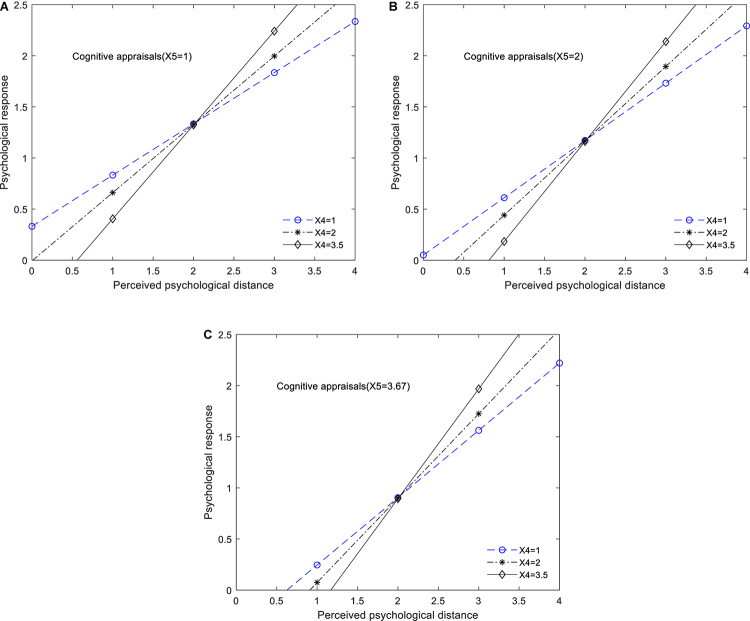
**(A)** The moderating effect of *perceived psychological distance* and *perception* of *justice enforceability* on the relations between social members’ perceived psychological distance and their *psychological response to PEBs adoption of other social members* (*cognitive appraisals* = *1*). **(B)** The moderating effect of *perceived psychological distance* and *perception* of *justice enforceability* on the relations between social members’ perceived psychological distance and their *psychological response to PEBs adoption of other social members* (*cognitive appraisals* = *2*). **(C)** The moderating effect of *perceived psychological distance* and *perception* of *justice enforceability* on the relations between social members’ perceived psychological distance and their *psychological response to PEBs adoption of other social members* (*cognitive appraisals* = *3.67*).

Figure 4 display the visualizing moderating effects of *perception* of *justice enforceability* and *cognitive appraisals*. In this study, we use the 16th, 50th, and 84th percentiles of the distribution of X4 (*perception* of *justice enforceability*). These are 1, 2, and 3.5, respectively. In the same vein, the 16th, 50th, and 84th percentiles of the distribution of X5 (*cognitive appraisals*) are 1, 2, and 3.67, respectively. From the above three figures, we could clearly observe that as the value of X4 increases, the effect of social members’ perceived psychological distance (X1) on their psychological response (X2) to others’ adoption of PEBs also increases. Besides, we can see from Figure 4 that as the value of *cognitive appraisals* gradually increase (X5 = 1, 2, 3.67, respectively), the slope of the line in Figure 4 become much steeper, although the moderating effect of *cognitive appraisals* is not significant, which suggest that with the value of *cognitive appraisals increasing*, the impact of perceived psychological distance on social members’ psychological responses (X2) to others’ adoption of PEBs will be further amplified.

## Discussion

The purpose of this study is to investigate the conditions on which the social members’ perception of psychological distance in a social system affects their positive psychological response to others’ adoption of PEBs. By testing the hypotheses with the data collected by survey, we find that in a social system, when the government cannot force social members to adopt PEBs through compulsory means, social members’ perceived psychological distance to environment change plays a key role in driving the adoption of the PEBs. Because social members engagement with PEBs need to pay extra costs, when only a few members but not most members engage in PEB, this will create an unfair atmosphere in which those social members who have adopted PEBs will have a sense of unfairness, thereby reducing their willingness to adopt PEBs. The results of the study show that social members’ willingness to adopt and spread PEB could be strengthened by enhancing their *perception* of *justice enforceability*, even under the condition that in their adoption of PEB they need to pay additional costs. In the following, we will focus on the theoretical and practical implications of this research.

### Theoretical Implications

Our theoretical model clarifies the mechanism by which social members’ perceived psychological distance affects their willingness to spread PEBs. Scholars have explored the spread of behaviors from the structure of social networks. In this study, we study the spread of behaviors, from some non-network structure parameters, that need people to pay extra costs. Existing studies suggested that network structure parameters, such as network connectivity, network distance, network density, and network centrality, are important factors that affect the spread of behaviors. Our research demonstrated that social members’ perceived psychological distances, their psychological responses to others’ adoption of PEBs can also affect their willingness to spread PEBs.

Further, our research found that social members’ perception of justice enforceability is a condition under which their perceived psychological distances affect their psychological responses to other social members’ adoption of PEBs. From the perspective of social comparison and social fairness, the deterioration of environmental problems will have a direct or indirect impact on all people in the world. Therefore, everyone has a responsibility to protect the environment. In the social system, moral standards will make people feel that it is unethical to not adopt PEBs, although doing so is not illegal, they are still willing to bear the responsibility of adopting PEBs. When a social member observes that other social members have adopted PEBs and made contributions to the protection of the environment, this will result in the effect of spillover and a social comparison, which will also encourage him to adopt PEBs, otherwise, he himself could have a sense of injustice. We know that injustice mainly coming from social comparison is an important factor motivating a person’s social behavior.

Finally, our work fleshes out the research on the spread of behavior in the social system. Our research found that social members’ psychological distance can decrease their willingness to spread PEBs through the mediating role played by their psychological reaction to other people’s adoption of PEBs. When the social members perceived they had a proximal psychological distance to environmental changes, they had a more positive psychological response to other social members engaging in PEBs, and their willingness to spread PEBs will also increase.

### Practical Implications

Our research suggests that when we face the deterioration of environment changes, and the objective network distance is not conducive to the spread of PEBs, or when social members need to pay more additional costs for PEBs, then we can enhance their willingness to spread PEBs by changing their perceived psychological distance toward environment changes. Because it is difficult for us to change the objective distance of social networks in reality, but it is much easier for us to change the psychological distance that social members perceive. We suggest that social members engaging in PEBs can also have some negative impacts on and bring an extra burden to their daily lives. Additionally, China is not actually the country with the most serious environmental problems in the world. Thus, social members in China have a distal perceived psychological distance to environmental changes, their enthusiasm for engaging in PEBs is not high. However, from a long-term perspective, environmental degradation will have a very serious negative impact on China’s economy and society, so protecting the environment must not be delayed. In this context, by changing the social members’ distance perceptions of environmental changes, their willingness to spread PEBs can increase, the implementation of environmental protection measures can be promoted, and a healthier and safer environment for future generations can created.

Our research shows that in reality, by raising social members’ awareness of environmental changes, their positive attitudes to environmental issues can be strengthened too. As China gradually enters a well-off society, people’ demands for a healthy and safe environment is enhanced too. At present, Chinese people’s awareness of environmental protection is gradually increasing, but their environmental awareness has not been completely transformed into real PEBs. Whether the environmental protection initiative is issued by the government or advocated by NGOs, its implementation will still encounter resistance in reality. In reality, perhaps people deem that environmental protection is very important, but they may not actually engage in PEBs in reality. Many people have the idea that protecting the environment is the business of other people, not their own. They think that the deterioration of the environment may harm other people rather than themselves. Therefore, when the government makes an effort to implement environmental protection policies, it is important to change the attitude of social members toward the issue of environmental protection, so as to amplify the effect of their psychological distance on the spread of PEBs.

Besides, our research findings can provide support for the government promoting the implementation of environmental protection policies. Our research shows that the abstract perception of psychological distance can change social members’ psychological responses to other people’s adoption of pro-environment behaviors, this depends on their perception of justice enforceability. If the government can create a fair environment for implementing environmental protection initiatives or policies, then the more proximal the psychological distance of environmental issues are perceived to be by social members, the more positive psychological reaction they will have to others’ adoption of PEBs, and their own willingness to adopt PEBs also is enhanced.

### Limitations of This Study and Further Research

Most studies have limitations, and this study is no exception. In this study, although we have verified from the perspective of fairness that the effect of social members’ perceived psychological distance on the spread of PEB that needs them to pay extra costs depends on the perception of justice enforceability and cognitive appraisals, yet we do not accurately measure the magnitude of the dependence. In addition, although our research shows that social members’ perceived psychological distance affects their response to other social members’ adoption of PEBs, yet we could not definitely claim that such an effect is linear or non-linear. So, the above questions can be the focus of future research. In the process of data collection, the questionnaires were distributed to residents who have lived in a city for more than 3 years, doing so may cause sampling bias. Because the length of time residents live in a city may affect their perceived psychological distance to the environmental protection of a city. In addition, our questionnaires are distributed at different times, which may also invoke sample bias.

## Conclusion

By our research findings, we suggest that the government can use social members’ perceived psychological distance as an effective tool to enhance the spread of PEBs. However, our conceptual model highlights the need to consider the contingency of the impact of social members’ perceived psychological distance on their response to other social members’ adoption of PEBs. Our research reveals that the impact of social members’ perceived psychological distance on their response to other social members’ adoption of PEBs is contingent on their perception of justice enforceability and cognitive appraisals (not significant) toward the adoption of PEBs. This research finding thus contributes to theory refining of the spread of behaviors and provides implications and recommendations about the implementation of environmental protection policies for the government.

## Data Availability Statement

The raw data supporting the conclusions of this article will be made available by the authors, without undue reservation.

## Ethics Statement

The studies involving human participants were reviewed and approved by the ethics committee of China University of Mining & Technology. The ethics committee waived the requirement of written informed consent for participation.

## Author Contributions

ZX was responsible for reviewing the literature and doing statistical analysis. QC contributed to manuscript drafting and helped to collect data. SL contributed to conventionalizing the core constructs and design of the study. All authors contributed to the article and approved the submitted version.

## Conflict of Interest

The authors declare that the research was conducted in the absence of any commercial or financial relationships that could be construed as a potential conflict of interest.
